# Why some do but most don't. Barriers and enablers to engaging low-income groups in physical activity programmes: a mixed methods study

**DOI:** 10.1186/1471-2458-11-507

**Published:** 2011-06-28

**Authors:** Janet Withall, Russell Jago, Kenneth R Fox

**Affiliations:** 1 Centre for Exercise, Nutrition and Health Sciences, School for Policy Studies, University of Bristol, Bristol, BS8 1TZ, UK

## Abstract

**Background:**

The beneficial effect of physical activity for the prevention of a range of chronic diseases is widely acknowledged. These chronic conditions are most pronounced in economically disadvantaged groups where physical activity levels are consistently lower, yet this group is particularly difficult to recruit and retain in physical activity programmes. This study examined the perceptions of participants, non-participants, and exercise leaders in a low-income area regarding barriers, motives, and enabling factors for organised physical activity with a view to improving recruitment and retention.

**Methods:**

A mixed methods research approach was adopted to guide data collection and analysis. A survey, incorporating the Motivation for Physical Activity Measure - Revised (MPAM-R), was used to assess the motivations of 152 physical activity session participants in a highly deprived suburban neighbourhood. The MPAM-R data were analysed using t tests, analyses of variance to estimate age, body mass index, and activity mode differences and Pearson's correlation coefficient to address associations. Semi-structured interviews were also conducted with 33 local residents who did not participate in activity sessions and with 14 activity session leaders. All interviews were audio-taped, transcribed verbatim and analyzed using an inductive thematic approach.

**Results:**

Participants reported cost, childcare, lack of time and low awareness as barriers to joining activity classes. The need for support, confidence and competence in order to take up activity was widely expressed, particularly among women. Once people are active, high levels of social interaction, interest and enjoyment are associated with improved levels of retention, with different types of physical activity scoring differently on these factors.

**Conclusions:**

This study suggests that some factors such as cost, the fear of 'walking in alone', accessibility of facilities, and appropriate communication strategies may be of particular importance to increasing recruitment of low income groups. Interventions targeting this group should consider low cost sessions and childcare; activities popular with the target group and associated with good recruitment and retention; sessions held at accessible times; a focus on fun and socialising; well-researched and designed communications strategies; targeting of friendship groups; clearly branded beginners' sessions, and the potential of social marketing as strategies. The evidence presented here suggests that the current UK government approach designed to 'enable and guide people's choices' may not be sufficient if low-income groups are to be effectively supported in changing their health behaviours.

## Background

The beneficial effects of physical activity for the prevention of a range of chronic diseases are widely acknowledged [1] and the need to increase public levels of health-enhancing activity underpins key elements of the public health policy in England and Wales [2]. Physical activity is associated with reduced risk of cardiovascular disease, type 2 diabetes, breast cancer, colon cancer, obesity, [3] depression [4] and dementia [5]. These chronic conditions are most pronounced in economically disadvantaged groups [6] where physical activity levels are consistently lower [7].

Economically disadvantaged groups are also less likely to engage with physical activity interventions [8], and interventions designed to change individual health behaviours are most likely to be taken up by participants who are white, middle class and female[9]. Even interventions directly targeted at disadvantaged groups are less effective in engaging ethnic minority or low-income populations [10]. For example, the Walking the way to Health initiative specifically targeted those who took little exercise and/or lived in areas of poor health, yet largely recruited relatively educated and affluent participants [11]. These reviews reveal that many attempts to improve public health are unlikely to be successful in reducing health inequalities.

The literature relating to participation in physical activity is substantial. Factors that are consistently associated with physical activity behaviour include past exercise behaviours; perceived self-efficacy; social support; self-confidence; access to facilities; physical environment, gender and socio-economic status [12,13]. Motivations to engage in physical activity often relate to physical and mental health, weight management and fitness while exercise adherence is more often associated with enjoyment, interest and social interaction [14,15]. A number of theoretical models have been studied in an attempt to explain, predict and ultimately change health behaviours. Some link attitudes, normative beliefs, intentions and levels of physical activity (Theories of Reasoned Action and Planned Behaviour) with only limited success, some focus on the stages and processes of change (The Transtheoretical model) [13,16,17]; while others seek to understand the apparent tension between the individual's positive attitudes towards engaging in physical activity and their inclination towards passivity, and the influence of intrinsic and extrinsic motivations (Self Determination Theory) [18].

There has been limited focus on the physical activity levels of low socioeconomic individuals, a group in the greatest need of change [12,13]. Furthermore, much of the existing literature has focused on the mechanisms of successful recruitment into research trials, and has not examined the specific processes of how participants, particularly those in low-income groups, might be effectively recruited into health promotion programmes. Where studies exist, they are often restricted to the difficulties of increasing representation of minorities into research [19]. The implications of enrolling in research studies which requires dealing with complex paper work including informed consent and data protection statements, and the possibility of being assigned to control groups are quite different. Such recruitment is also affected by factors specific to involvement in trials such as mistrust of research and the medical system, perceived harms of trial participation, the associated time and financial costs and fear [20] which are not necessarily relevant to recruitment into health promotion interventions. The effectiveness of recruitment and retention strategies affect the success of every field intervention designed to reduce health inequalities, yet the literature available to guide practitioners is therefore limited.

Many community activity programmes are funded but rarely fully evaluated and problems of poor recruitment and retention rates are common [9]. In order to maximise the health benefits and return on investment of these programmes, and to facilitate their sustainability, recruitment and retention rates need to be increased. To achieve this we need to know which people do and do not attend, their reasons why, and how this information can be used to design successful interventions. In order for effective recruitment strategies to be developed the difficulties of engaging low-income groups in health promotion programmes needs to be fully understood in its broadest sense, rather than as an adjunct to specific individual trials or pilots.

To address the research gaps identified above, this study examined the factors that affect low-income groups' participation in organised physical activity from the perspective of participants, non-participants and activity session leaders in order to understand the barriers and facilitators for both adoption and retention to activity programmes in a high deprivation area in a large city. A mixed methods approach was used to provide an insight that took account of the views of the key stakeholders [21].

## Methods

### Setting

The selected study area was Southmead, a suburb with the lowest life expectancy (75.3 years) in the city of Bristol (UK), which is categorised by postcodes with the BS10 prefix [22]. Using the Index of Multiple Deprivation, a UK government produced area level measure of deprivation that includes assessments of income, employment, health and education [23], Southmead Central is one of the 1% most deprived areas in England [24]. Across the ward 26.8% of the population suffer from income deprivation, rising to 49.5% in central areas, with 66.7% of children affected by poverty. Despite priority investment to increase physical activity [25] the area has a significantly below average percentage of residents who exercise at least once a week [26]. The area has a small leisure centre converted from old school buildings.

### Mixed methods research

Issues relating to participation in physical activity are complex. Utilizing the combined strengths of both qualitative and quantitative approaches offers the best possible approach to building a fuller insight into an issue that has little previous evidence to guide research. While a quantitative approach can reveal the attitudes, beliefs and experience of physical activity, qualitative techniques allow enquiry into how and why people do or do not engage and adhere. As such a combined approach can provide an understanding that might be missed through using only a single method [21]. Mixed methods can strengthen evidence through confirmation and substantiation of findings while the different data collection methods can neutralise each others' biases or weaknesses [27].

### Procedures

The study was comprised of three interlinked components: Component 1 was a survey of participants at physical activity sessions (n = 152). Component 2 was a series of semi-structured interviews with people who did not participate in physical activity sessions (n = 33). Components 1 and 2 allowed a comparison of the motivations, enablers and barriers to exercise encountered by this low-income group and those reported in the literature, highlighting any factors particularly relevant to successful recruitment of this group. Component 3 involved semi-structured interviews with physical activity session leaders (n = 14). This component provided an external, observed perspective on the issues of recruitment and retention which could be compared with the findings of Component 2.

### Component 1

The first component was designed to identify the types of people who currently attend community programmes, their attendance patterns and the motivations that had successfully led to their accessing activity sessions. This phase comprised of a survey that asked age (under 18 yrs, 18-34 yrs, 35-54 yrs, 55+), gender (M, F), postcode (BS10, non-BS10), height (ft, ins or cms), weight (st, lbs or kilos), ethnicity (White, Black/Black British, Asian, Other), attendance duration (<1 mth, 1-3 mths, 4-6 mths, 6-12 mths, 1-2 yrs, 2 yrs+) regularity of attendance at community programme (most weeks, less often), attendance with a friend (Y, N), communications channel and attendance at other sessions or community groups (Y, N). Participants also completed the Motivation for Physical Activity Measure - Revised (MPAM-R). The MPAM-R assesses five motives for engaging in physical activity, appearance; social; competence; fitness; and interest/enjoyment [28]. This scale indicates the relative importance of these main motivators to exercise.

Recruitment into Component 1 began by identifying and contacting all of the physical activity session leaders in the study area. Where permission was given the researcher attended, explained the study and distributed information sheets to attendees. Written, informed consent was obtained prior to surveys being completed in private with a researcher on hand to assist where low literacy levels caused response difficulties.

### Component 2

The second component consisted of semi-structured interviews with 33 local residents. As barriers to recruitment and exercising are complex, Component 2 featured a qualitative approach to seek an in-depth understanding of the issues relating to recruitment and retention. Two interrelated issues exist here: 1) the barriers, enablers and motivations which relate to joining any group; and 2) the barriers, enablers and motivations which specifically relate to engaging specifically in physical activity taking place in a group setting. The intention here was to gain a unique perspective on, and separate out, the issues relating to both the 'joining' and the engaging in physical activity, and their relative importance. It is important to distinguish between these two elements as they are likely to require addressing in very different ways if recruitment and retention is to be effective.

Two groups of interview participants were recruited; 1) people who attended a non-physical activity group (joiners), and 2) people who did not attend any group at all (non-joiners). Using a purposive sampling method, 12 joiners were recruited from toddler groups, coffee groups and a choir and 21 non-joiners who were recruited at a local primary school and an advice centre. The researcher attended each venue, explained the study and distributed information sheets. Those willing to participate were interviewed at local community centres or in their own home using a guide designed to identify the key issues that affect participants' physical activity behaviours. The interview guide was piloted with two participants and refined before use in the main study. Introductory questions regarding leisure time activities preceded examination of the barriers to participating in activity sessions and potential enablers, awareness levels of local activity sessions, current promotional methods, media habits and communication and information channels. The 'joiners' were also asked about the process of joining their current group. The guides were developed to address the issues previously recorded in the literature such as cost, access to childcare, lack of time, availability and social support [13,15] while also allowing the participants to present their own views. Written, informed consent was obtained before all interviews. All interviewees also completed a short questionnaire to record gender, age, ethnicity and physical activity amounts and patterns. To avoid any literacy issues the questionnaire was completed jointly with a researcher. Interviews were digitally records and lasted between 20 and 45 minutes.

### Component 3

To provide an external, observed perspective on the issues of recruitment and retention Component 3 comprised of semi-structured interviews with 14 activity session leaders. Participants were recruited by contacting all those session leaders identified in Component 1. Session leader who were willing to take part were interviewed at local community centres or in their own home. Written, informed consent was obtained before all interviews and the interview guide was piloted with two participants and refined before use in the main study. Interviews were digitally recorded and lasted 20-40 minutes. The interview guide explored the perceived barriers, enablers and motivations to participate experienced by session attendees; and the factors affecting adherence. Session leaders' experiences of recruitment methods and challenges were also sought.

All three components of the research were approved by the University of Bristol School of Applied Community Health Studies Research Ethics Committee.

## Analyses

### Survey

Student t-tests were used to examine if motivations for exercise (MPAM-R) differed by area of residence (local/non-local), gender (male/female), duration of attendance and attendance with a friend. Analysis of variance tests, with paired comparison follow-ups tests were used to compare motivations across age group (under 18 yrs, 18-34 yrs, 35-54 yrs, 55+), activity type (aerobics, strength/flexibility, dance and sport) and BMI range (< 18.5, 18.5-24.9, 25+). A principle component factor analysis was performed on the 30 items in the MPAM-R measure to ensure the factor structure in this dataset was comparable to the originally derived factors. Pearson's correlation coefficient was used to examine associations between motivations to exercise (MPAM-R) and duration of attendance and attendance with a friend. All analyses were undertaken in SPSS (version 14.0) and alpha was set at p < 0.05.

### Interviews

All transcribed text was entered into NVivo Software for Qualitative Research Version 8. The text from the two phases was coded into separate databases by the researcher and a sample checked by a second researcher. Inductive thematic analysis was used to reveal the main themes and salient quotes that captured the essence of the themes were extracted. Given the emergent nature of the data no hypothesis or structures were applied although emergent themes were considered in the light of theories of motivation.

## Results

### Survey

The survey was piloted and refined prior to being completed by 152 participants at 22 different activity sessions. Participant characteristics are shown in Table 1.

A factor analysis revealed that the 30 items in the MPAM-R measure all loaded above .613 on their hypothesised factors, and alpha scores for each subscale were .88 for interest/enjoyment, .88 for competence, .86 for appearance, .89 for fitness and .84 for social.

**Table 1:** Characteristics of the survey study population (n152)

Demographic characteristics	Total N	Total %
Participants reporting postcode (n144) living	144	100
Within study area	69	45.4
Outside study area	75	49.3

Age (n140)		
<18 years	23	16.4
18-34 years	20	14.3
35-54 years	15	10.7
55 years +	82	58.6

Gender (n144)		
Male	32	22.2
Female	112	77.8

BMI (n121)		
Underweight	6	5
Normal weight	60	49.6
Overweight/obese	55	45.5

Ethnicity (n139)		
White	126	90.6
Black/Afro-Caribbean	7	5
Asian	2	1.4
Other	4	2.9

Associations between factors on the MPAM-R measure are summarised in Table 2. There was a weak positive association between attending with a friend and high scores on the interest/enjoyment (r = .203, p = .019) and the social subscale (r = .191, p = .028). Duration (attendance for greater than 3 months) was positively associated with high scores on the interest/enjoyment (r = .306, p = .000) and the social subscale (r = .201, p = .020), and negatively associated with appearance as a motivator (r = -.241, p = .007).

**Table 2:** Correlations between motivations to exercise on the MPAM-R scale and attending sessions with a friend and extended duration of attendance (>3 mths)

	With a friend	Duration
Interest/Enjoyment	.203*	.306**

Social	.191*	.201*

Appearance	.104	-.241**

Competence	-.005	.084

Fitness	-.022	.036

*Significant at 0.05 level (2 tailed)

**Significant at 0.01 level (2 tailed)

There were significant differences between the 55 yrs+ age group and all other age groups on the social subscale (*p *= .023, *p *= .005, *p *= .040) with the older age group more motivated by this factor. There were differences between the two middle age ranges (18-34 yrs and 35-54 yrs) and both the younger (<18 yrs) (*p *= .015, *p *= .024) and older ranges (55 yrs+) (*p *= .001, *p *= .006) who were motivated more by interest/enjoyment (Table 3). Strength/flexibility session participants were more motivated by interest/enjoyment than those in aerobic sessions (*p *= .016) and dancers more than those in aerobic or sport sessions (*p *= .000, *p *= .004). Participants in dance and strength/flexibility sessions were more motivated by social interaction than those in aerobics sessions (*p *= .012, *p *= .013) as were those in strength/flexibility sessions compared to sport (*p *= .003). Fitness was a greater motivator for those in aerobics and strength/flexibility sessions compared to those engaged in sports (*p *= .000) (*p *= .003) Motivation based on appearance was also higher among aerobics participants compared to those engaged in dance or sport (*p *= .010) (*p *= .001) (Table 4).

**Table 3:** Motivations for exercise as assessed by the MPAM-R scale by different age group (n148)

	Age group 1 <18 yrs (*N *= 24)	Age group 2 18-34 yrs (*N *= 23)	Age group 3 35-54 yrs (*N *= 16)	Age group 4 55 yrs+ (*N *= 85)
	**Mean (SD)**			

Interest/Enjoyment	5.77 (0.96)^a, b^	4.77 (1.63)^a, d^	4.89 (1.35)^b, e^	5.85 (1.15)^d, e^

Social	3.93 (1.20)^c^	3.67 (1.50)^d^	3.85 (1.54)^e^	4.80 (1.61)^c, d, e^

Appearance	4.98 (1.12)^c^	5.04 (1.52)^d^	4.69 (1.10)	3.99 (1.79)^c, d^

Competence	5.58 (1.12)	5.16 (1.56)	5.46 (1.21)	5.17 (1.53)

Fitness	5.96 (0.82)	5.87 (1.15)	6.18 (0.75)	6.15 (1.29)

Superscripts (^a, b, c, d, e^) indicate mean difference in pairwise comparisons (p < 0.05)

^a ^= Group 1 v Group 2, ^b ^= Group 1 v Group 3, ^c ^= Group 1 v Group 4,

^d ^= Group 2 v Group 4, ^e ^= Group 3 v Group 4

**Table 4:** Motivations for exercise as assessed by the MPAM-R scale by different activity type (n152)

	Activity type 1 Aerobics (*N *= 58)	Activity type 2 Strength/Flex (*N *= 29)	Activity type 3 Dance (*N *= 30)	Activity type 4 Sport (*N *= 35)
	**Mean (SD)**			

Interest/Enjoyment	5.07 (1.50)^a, b^	5.99 (0.83)^a^	6.23 (0.85)^b, e^	5.36 (1.34)^e^

Social	3.89 (1.77)^a, b^	5.01 (1.41)^a, d^	4.87 (1.53)^b^	4.20 (1.14)^d^

Appearance	4.95 (1.42)^b, c^	4.58 (1.64)	4.00 (1.78)^b^	3.78 (1.57)^c^

Competence	5.30 (1.63)	5.52 (1.13)	5.60 (1.12)^e^	4.83 (1.40)^e^

Fitness	6.32 (0.88)^c^	6.48 (0.67)^d^	5.91 (1.29)	5.31 (1.50)^c, d^

Superscripts (^a, b, c, d,e^) indicate significant mean difference in pairwise comparisons (p < 0.05)

^a ^= Group 1 v Group 2, ^b ^= Group 1 v Group 3, ^c ^= Group 1 v Group 4,

^d ^= Group 2 v Group 4, ^e ^= Group 3 v Group 4

### Interviews

A summary of the characteristics of the participants who took part in the Component 2 interviews is presented in Table 5. All participants lived within the designated economically-disadvantaged area (Southmead, Bristol) and the majority were white, a reflection of the predominant ethnicity of the area. The 14 session leaders in Component 3 interviewed represented 17 of the 29 different types of session available in the area.

**Table 5:** Characteristics of interview participants (n33)

	Group members	Non group members
Male	1	9
Female	11	12

18-24 yrs	0	3
25-34 yrs	2	6
35-44 yrs	5	6
44-54 yrs	4	5
55 yrs +	1	1

White/Caucasian	12	19
Asian	0	1
Black British	0	1
Other	0	0

Meet exercise recommendations (30 mins a day, 5 times a week)	0	3
Yes	12	18
No		

Table 6 summarizes the key themes from the Component 2 interviews indicating interviewees' attitudes and motivations to exercise; reasons for not participating in organised activity sessions; key enablers that may support them in overcoming these barriers and mechanisms associated with session awareness. The same table shows the key themes from Component 3 interviews i.e. the session leaders' perceptions of the same issues.

**Table 6:** Key themes from interviews (n = 47)

	Interviews with session leaders/initiators	Interviews with local residents
**Attitudes**		Good awareness of benefits of exercise

		Attitudes largely positive but some negative and some feelings of exercise being irrelevant

**Motivations**	weight issues and physical health particularly in older adults	weight issues, physical and mental health, fitness

	socialising and enjoyment	

**Barriers**	lack of confidence	lack of confidence

	fear of stepping into an activity session alone	reluctance to attend alone

	perceived lack of competence	perceived lack of competence

	Cost	Cost

		requirement for and concerns regarding childcare

		low priority, issues of time and work patterns

**Enablers**	fun, friendship and socialising	attending with a friend

	integration of sessions into the community	self confidence

		Low cost, good availability

**Awareness**		good awareness of benefits of exercise

		patchy awareness of available sessions

	power of networking and personal contact as recruitment tool	power of word-of-mouth as a communications channel

### Perceptions of physical activity

Almost all interviewees revealed awareness of the benefits of being physically active. However, there appeared to be no links between this and participation in activity.

'I think it's really, really important to be healthy and active but it's not like the be all and end all ...'(Female, 35-44 yrs)

Some felt that exercise was not something that related to them,

'My partner sort of found out about the gym ... but I've never really thought of anything for me'. (Female, 35-44 yrs)

while others viewed it as a low priority, or not something they enjoyed.

'I'd rather be doing something else. I know that sounds awful ......' (Female, 45-54 yrs)

Many people appeared to have very positive attitudes to exercise,

'I used to do keep fit a couple of years ago. It was great, you feel active, you feel fit'

'I think there's a gym .... I'm going to find out how much it is to join and get back involved' (Male, 25-34 yrs)

and being active did not appear to be out of line with local social norms.

'I don't have any limitations of what I do off family and friends' (Female, 45-54 yrs)

Preferred activities varied from gentle activities for older adults and those with health problems to swimming; different types of dance; the gym and aerobics; with some people interested in more challenging activities such as outward bound or assault courses.

### Motivations to exercise

Positive effects on mental well-being were mentioned almost as often as those on physical well-being.

'I want to make sure I'm healthier for my kids, that I don't get ill from my diabetes or anything, if I don't control my weight properly'(Female, 25-34 yrs)

'I get mental illness as well so the gym is very good for me'. (Female, 35-44 yrs)

Some were motivated at least partially by a wish to be 'fit' but in association with good health or being able to keep up with young children.

'Yeah just to get fitter so that I can run around with the kids at football, instead of dying just carrying the water bottles'(Female, 35-44 yrs)

Wanting to lose weight was very closely associated with exercise for women and for some men.

'I was very slim before I had children and didn't see the point in bothering to do it'

'I need to exercise to get rid of some of the weight' (Female, 25-34 yrs)

'Well obviously I started doing that in general to lose weight'(Female, 18-24 yrs)

Overweight for some was related closely to physical health whereas for others it was wholly about appearance.

A large number of people had been active in the past. Cost, change in lifestyle or work patterns, lack of time and enjoyment were the other main factors cited as negatively affecting on-going participation.

'I just couldn't afford it. Every time I thought oh I'll go put some money on my card and then it's like oh no I've got to buy nappies, oh no I've got to do this, oh no the phone bill needs paying'(Female, 18-24 yrs)

'but I get fed up and so I give up.... you go there and it's just boring'(Female, 45-54 yrs)

Session leaders' perceptions of motivations to exercise were slightly different to those of participants. So central was weight as a motivator that some session leaders perceived that not being overweight was a serious disincentive.

'Quite a few of the ones that are really difficult are the ones that aren't quite overweight' (Session leader)

Few local people discussed enjoyment or socialising as motivations to exercise while most of the session leaders believed that both had a substantial impact on participant retention.

'They come for a giggle, for a chat, they come to have fun and keeping fit is almost by the by really' (Session leader)

### Barriers to starting activity

Cost is very commonly perceived to be a major barrier to initiating activity,

'I really do enjoy exercise and activity but the only other reason I don't do it is moneywise'. (Female, 35-44 yrs)

and was the most common cause of dropping out of sessions.

'...it was only £17 but Christmas, kids and all that'. (Female, 35-44 yrs)

Not wanting to attend alone was a prominent barrier, particularly amongst women.

'I don't like going places on my own.... I don't like mixing unless I've got someone to talk to'. (Female, 45-54 yrs)

'Wouldn't go on my own' (Female, 35-44 yrs)

'I'd do it. I'd have to have someone go with' (Female, 45-54 yrs)

It was associated with lack of confidence,

'If I was to come on my own I don't think I would because I'm not a very confident person'. (Female, 25-34 yrs)

This was something that was also observed and acknowledged by a number of session leaders.

Childcare was an issue although the cost of a creche and a reluctance to leave children with strangers were also concerns for some.

'I would go to a gym or fitness classes, if I knew if I could get someone to have the children. I would do it, I would' (Female, 25-34 yrs)

'I would feel wary about my little ones with someone I didn't really know' (Female, 25-34 yrs)

Time was a commonly referenced issue particularly relevant to employed men and those with large families. Session times often made them difficult to fit into life and work patterns.

'...so there just aren't enough hours in the day'(Female, 35-44 yrs)

'You get home and tidy up after eight, it leaves not a lot of time left'(Female, 35-44 yrs)

'I haven't got much spare time. I work and we're foster parents'(Male, 35-44 yrs)

Some potential participants felt restricted by health issues. Some were concerned about not being as competent or fit as existing attendees

'you just feel like you're pulling the group down and it's not enjoyable then'. (Female, 35-44 yrs)

'you tell yourself negative things. Oh there's people there who've been training for a while, they're all a lot fitter than you' (Male, 25-34 yrs)

Session leaders' perceptions of barriers to activity largely related to cost issues,

'if ... they're freezing cold and no track suit we ain't going to take a pound off them' (Session initiator)

though some had reservations about free sessions.

'a lot of communities get a lot of free courses and I do wonder about that. You know, the level of commitment'. (Session leader)

### Enablers of attending physical activity sessions

As attending alone presents a barrier, attending with a friend enables participation, but almost exclusively amongst females. This was discussed as a pre-requisite of future participation by those who were non-joiners,

'Like walking into a room with a lot of people you usually feel quite nervous. 'Coming along with a friend for support makes it easier' (Female, 45-54 yrs)

and had proven to be an enabler of current participation by those who had joined non-exercise groups.

'It is quite nervy walking in to an established group and thinking am I going to fit in but thankfully my neighbour asked me to come along so...' (Female, 35-44 yrs)

Having the confidence to attend, particularly alone, was more common in males, yet they still were not currently active, so other barriers remained.

'I think in general people like someone with them... myself I don't really care'. (Male, 35-44)

A variety of individuals from the session leader to friends and even professionals appear to be viable supporters of attendance.

'If maybe someone who was running the group came around to see me and said come along then a familiar face...that would be quite nice but...'' (Female, 35-44)

Only a few session leaders mentioned attending with a friend as a potential enabler directly but many of them referred to the appeal of the social aspects of attending.

'a lot of them just enjoy meeting up with their friends, having a laugh' (Session leader)

### Awareness of opportunities

Awareness of activity sessions was very patchy. People felt uninformed about what was available locally. The vast majority of participants, joiners and non-joiners, referred to word of mouth as their main source of information.

'everything is word of mouth....it is the biggest thing because you get a review of it then at the same time as hearing about it.' (Female, 25-34 yrs)

Some people read posters in public places and flyers that came through their doors and the internet was well used by some but not others, but it was often not considered to be a source of local information.

'I'd never think of using the internet for something round here' (Female, 18-24 yrs)

## Discussion

The purpose of this study was to increase understanding of participation, or non-participation, in physical activity programmes by low income groups, and so to inform design of strategies to increase programme recruitment and retention. This study revealed several barriers, enablers and motivations which are consistent with previous research [12,13,15], but that some of these factors have greater impact on the behaviour of low-income groups than other groups.

The key reported barriers were cost, access to childcare, lack of time and low awareness. Each of these presents practical barriers, but these are simple and straightforward concerns to articulate so it is possible they may mask other barriers to change. It is therefore not clear if engagement would increase if these barriers were addressed. In addition, perceptions lack of social support, and low perceived confidence and competence was widespread, particularly amongst women. Key enablers, largely revealed by those who had been or were currently active, were high levels of social interaction, interest and enjoyment. With the exception of concerns around competence, reported barriers, enablers and motivations to participate in exercise groups were very similar to those expressed by participants who had joined groups not related to exercise. Clearly, the difficulties people experience in joining a group, and barriers to participating in exercise, both need to be addressed, as both would affect recruitment and retention into physical activity sessions.

The UK Coalition government's recent Public Health White Paper targets health inequalities and advocates increasing choice and using a 'nudge' approach to behaviour change [29]. Amongst the many and varied issues involved in this complex decision-making and behaviour change process there are clearly many barriers to engagement in physical activity a simple positive nudge in terms of availability, information, and choice may not be sufficiently powerful on its own, to tip the balance, particularly for hard-to-reach and health needy populations. Such an approach appears to leave the psychological and social barriers that are particularly relevant in low-income groups unaddressed, and as such may widen health inequalities.

The cost to the individual of organised structured exercise activities has been associated with reduced participation [30,31]. Issues of cost have a proportionately greater impact on low-income groups. Where a more affluent individual can reprioritise exercise ahead of other leisure activities this is likely to be more difficult for people with less financial resource who would have to reallocate funds from what for them are more important or essential purchases. The groups in which 'joiners' currently participated were all free or very low cost. While cost presents such a prominent and easily articulated obstacle it is difficult to fully understand to what extent addressing cost issues would positively affect recruitment or whether other barriers would still prevent behaviour change.

Lack of childcare can be a greater barrier where a partner, or close family, may not be present and the cost of childcare is prohibitive, while some 'lack of time' concerns may relate to 'lack of child-free time'.

Social support has been shown to be positively associated with exercise levels [13] with low confidence and low self-efficacy negatively associated [14,15]. Interestingly this study extended these findings to show how a perceived lack of competence, low levels of self-confidence, and a powerful need for a sense of relatedness within a group situation, were often addressed through attending with a friend. This applied to those contemplating initiation and was strongly corroborated by those already attending a non-exercise related group.

### Enablers to exercise

Awareness of the health benefits of exercise was high and attitudes to exercising were largely positive. Though these factors do not necessarily lead to behaviour change they provide an important basis for individual change. The current government approach of 'guiding people's choices' may reinforce this awareness but offers little in support for the key step from knowledge to action [29].

Fun, enjoyment and socializing are commonly quoted motivations to exercise [14]. Session leaders' perception of participants' motivation supported this while participants who were not currently exercising reported fitness, weight-loss and health-related motivations. This may relate to session leaders observing the enjoyment and sociability occurring in a session, while non-attendees are not currently party to this experience. These different motivations underpinning recruitment and retention have been recognised [32], with initiation motivations relating to optimistic expectations of future outcomes of taking part while maintenance is more affected by the actual outcomes of the new behaviour [33]. For example, initial weight and health-related motives appear to quickly change to fun and socialising [15]. Though the survey provides only weak support for an association between attendance with a friend and long term participation, attendance with a friend is associated with high levels of interest, enjoyment and socialising, which in turn support retention. Interviews with joiners of non-exercise groups also strongly highlighted the positive impact on attendance and adherence of attending with a friend. A further consideration is that many commonly available activities such as aerobics and spin sessions appear to offer lower levels of enjoyment and sociability than dance and sport sessions and this may also have an impact on retention.

### Session awareness

There is no central source of information on session availability in the study area but thirty-seven weekly sessions were found to be available, so the perception of a lack of availability can be at least partially attributed to low awareness. Promotion was largely limited to posters, flyers, the local newspaper and word of mouth. Word of mouth was widely quoted as the most common source of knowledge regarding community activities. However, its apparent importance as a communications channel may be partially due to a lack of any effectively implemented alternative promotional strategy.

### Theoretical models

Although this research was not grounded in an individual theory of behaviour, it offers several findings that suggest a particular theoretical framework may apply to exercise behaviours in this population. In common with other studies [13], there was little support shown here for a link between attitudes, normative beliefs, intentions and levels of physical activity, which indicates that theories based on these constructs, such as that of Reasoned Action and Planned Behaviour, would have only partial application in understanding behaviour and underpinning behaviour change in this group [16,17]; rather the findings here link more closely with the tenets of Self Determination Theory (SDT). Recently applied in exercise and weight loss settings [34], SDT seeks to understand the degree to which the health behaviour contributes to satisfying the basic psychological needs of feeling competent, autonomous (in control as an agent of the behaviour) and relatedness or belonging[18].

Findings from this study support the substantial base of applied research that demonstrates that enjoyment, confidence, competence, intrinsic motivation, and autonomous regulation are consistently correlated with regular participation in physical activity [35-37]. The evidence also clearly supports SDT's premise that an individual's intrinsic motivational tendencies usually require external support to result in maintained behaviour change, and that these tendencies are easily reversed by a non-supportive environment [38]. In particular, the interviews with non-exercise session participants, clearly indicated the influence of support and a sense of relatedness within a group (attending with a friend, influence of peers) that was required to enable participation. The power of the influences beyond the self is clearly evident.

### Implications for recruitment and retention

Many in the target group wee aware that being physically active is beneficial to physical and mental health, and many expressed a positive attitude towards engaging in organised activity, but were not currently doing so. Other issues are clearly at play, and it is these issues that need to be addressed if recruitment, and ultimately retention, rates are to be improved. The key issues revealed by the three components of this study; potential solutions to these issues that were suggested by participants or implied by findings and potential challenges that may need to be addressed if these solutions are to be implemented are all outlined in Table 7 and discussed below.

**Table 7:** Issues for recruitment and retention from Components 1, 2 and 3

Issues affecting recruitment and retention	Potential solutions stated by participants or implied by findings	Potential challenges relating to solutions
Cost	Low cost/free sessions	Unsustainability. May mask other barriers

Childcare	Low cost, accessible childcare	Unsustainability. May mask other barriers

Low confidence	Use of friends/contacts as support. Recruit via pre-existing group, venue or target friendship groups	Difficult to reach more isolated individuals

Low perceived competence	Provide clearly branded beginners session	Participants will become more competent - may disincentivise those who join later

Lack of time	Accessible sessions at times to suit the target group	May require provision of activities at a wide range of times

Enjoyment	Consumer research into, and provision of, preferred activities	May require provision of wide variety of activities

	Focus on those associated with enjoyment and sociability	

Session awareness	Investment in good communications strategies	Up front and on-going investment, expertise and commitment required

Initial health and weight related motivations	Initial recruitment to include appeals to these motivations, sessions to deliver enjoyment and sociability to aid retention	On-going challenge to maintain enjoyment as initial enthusiasm declines - and across a mixed ability group

Targeted interventions	Research and Segmentation. Low income groups are not amorphous - an understanding of key sub groups is required to underpin effective interventions	Time consuming and expensive

The impact of addressing issues of cost by offering very low cost sessions, as part of a well-designed intervention, has not been thoroughly tested. Therefore although there are issues about the long-term sustainability of subsidised approaches there is a need for research to examine if such campaigns can work. The need for individuals to shift priorities between exercise and other competing activities and the relatively greater demands on financial resources, requires even more effective strategies to enhance the attractiveness of the benefits that distinguish the desired behaviour from competing activities [39].

It may be possible, to some extent, to circumvent the very complex issues relating to self-confidence, by attracting the attendance of existing friendship groups. Recruiting from other established groups or at a venue where people have already gathered, such as a children's centre or the school gates, may help eliminate the fear of 'walking in alone' and provide continued support for attendance.

Clearly branded beginners' sessions which require no expertise, experience or fitness, where participants are all starting out together, could go some way to tackling issues of self-efficacy and competence. Practically this may be effective for early joiners of a programme but after a period these 'beginners' may have developed levels of competence which deter new participants.

Session timing should be carefully considered so that competing demands on time are minimised. For example in target groups where full-time employment levels are lower, daytime sessions may be more accessible. Sessions aimed at mothers could follow directly from the school run; an additional journey out of the house is not required and other daily activities are only delayed by the minimum amount of time.

Formative research with the target group, provision of appropriate activities and good availability enable choice so that participants can opt for an activity they will enjoy and can access. However it is important to consider the different motivations involved in initial participation (weight and health issues) and retention (enjoyment and sociability) and ensure each is fully addressed at the appropriate stage.

In low-income populations, where issues of literacy and low reading culture are more likely to be present, properly targeted and well-designed communications strategies specifically addressing these issues, and generating and supporting word of mouth promotion, may be particularly important. Such a communications strategy would require a reasonable start-up and ongoing financial investment.

Noticeably this, and many of the other issues outlined above, could effectively be addressed within the structure of a social marketing approach. Over recent years social marketing techniques have shown promise as a means of improving the development and promotion of health programmes [40] and are now supported by UK government policy [41]. The central concept of the 'marketing mix', also defined as the 4 Ps (product, price, place and promotion), focuses on the development of the most appealing product at the right price, delivered at an accessible and appealing place (and time) and effectively promoted, all underpinned by thorough consumer research and effective segmentation [42]. However although community level social marketing interventions have been developed, implemented and reported these have largely been non-academic studies and have lacked rigorous analysis, evaluation and published results [43]. It is an approach that appears to have potential in increasing recruitment and retention of low-income groups but further research into its effectiveness at a community level is required.

The evidence presented here suggests that a government strategy designed to go further than 'enabling and guiding people's choices' will be required if low-income groups are to be effectively supported in changing their health behaviours [29].

## Limitations

This study adds to the existing literature in that relatively few mixed methods studies have specifically focused on barriers, enablers and motivations to participating in organised physical activity sessions in a low-income community. The triangulation of the data enabled the issues to be viewed from an internal (local residents) and an external view (session deliverers), and pre and post engagement in activity, giving a deeper understanding of the influences at work and corroborating the interpretation of the data. This approach improves the validity of the data and increases its comprehensiveness [44]. The study does however have some limitations. Only organized activity sessions were included and physical activity undertaken individually was not studied. The small number of non-white participants meant that views of individuals from BME groups were not well represented, however this reflects the predominant ethnicity of the area with BME residents making up only 5.2% of the population [26]. The purposive sampling strategy in the qualitative arm of the study may have impacted the representativeness of the participants. Men were difficult to recruit and are somewhat underrepresented in the qualitative sample.

## Conclusions

This study suggests that there are some key issues relating to increasing recruitment and retention into physical activity sessions that have a greater impact on low-income groups than the general population; and that barriers relate to both joining a group and participating in exercise. These include practical barriers such as cost and childcare; communication issues relating to session awareness; the specific support required by women to attend organised exercise sessions; how the attractiveness of exercise can be enhanced and its priority increased; and issues of perceived competence. In order to address these issues interventions designed to increase and maintain participation should consider low cost sessions and childcare; activities popular with the target group and associated with good recruitment and retention; sessions held at accessible times; a focus on fun and socialising; well-researched and designed communications strategies; targeting of friendship groups and clearly branded beginners' sessions. It appears that SDT may provide a useful theoretical framework through which to study the motivations and behaviour of this population, and a basis of interventions to tackle barriers to recruitment. Social marketing techniques may provide a suitable framework for such interventions. Further research is required to assess whether these approaches would positively impact recruitment and retention of low-income groups into exercise sessions.

## Competing interests

The authors declare that they have no competing interests.

## Authors' contributions

The study was designed by JW, RJ and KF. Analysis was performed by JW. The first draft of the paper was written by JW and all authors provided critical input and revisions of all further drafts.

## Pre-publication history

The pre-publication history for this paper can be accessed here:

http://www.biomedcentral.com/1471-2458/11/507/prepub
